# Paediatric tibial tubercle fractures – A retrospective case series

**DOI:** 10.1371/journal.pone.0355958

**Published:** 2026-08-13

**Authors:** Maarten H. W. Mosch, Albert F. Pull ter Gunne, Sjoerd R. J. Jens, Corné J. M. van Loon, Edo J. Hekma, Diederik P. J. Smeeïng

**Affiliations:** 1 Department of orthopedic surgery, Rijnstate hospital, The Netherlands; 2 Department of trauma surgery, Rijnstate hospital, The Netherlands; 3 Department of radiology, Rijnstate hospital, The Netherlands; Università degli Studi del Piemonte Orientale Amedeo Avogadro: Universita degli Studi del Piemonte Orientale Amedeo Avogadro, ITALY

## Abstract

**Background:**

Tibial tubercle fractures (TTFs) are rare injuries in adolescents, representing 3% of all proximal tibia fractures and less than 1% of all physeal fractures. Despite their low incidence, they can be associated with significant complications such as periosteal stripping, vascular compromise, compartment syndrome and intra-articular damage. Given the increasing incidence due to youth sports participation and the limited existing literature, this study aimed to describe patient characteristics, treatment methods, outcomes, and complications in children aged 8–16 years with TTFs.

**Method and findings:**

A retrospective cohort study was conducted at a level 2 trauma centre in the Netherlands, including 40 patients (41 fractures) between 2008 and 2023. The mean age at injury was 14.3 years, and 95% (38/40) of cases occurred in males, predominantly during sports activities. Thirty-two fractures (78% (31/41)) required surgical treatment, most commonly with screw fixation, while nine fractures (22% (9/41)) were treated non-operatively. The total complication rate was 9.8% (4/41), including two refractures and one implant loosening, all within the surgically treated group. No compartment syndromes were reported. At the end of follow-up 93.5% (29/31) of surgically treated patients and 77.8% (7/9) of non-surgically treated patients had returned to sport.

**Conclusion:**

Tibial tubercle fractures in adolescents occur mostly in males during sports. The surgical treatment is not standardized and can be custom made per fracture. Patients receiving surgical or non-surgical treatment both have a good chance to return to sport after this specific injury.

## Background

The tibial tubercle fractures (TTFs) represent 3% of all proximal tibia fractures and less than 1% of all physeal fractures in the adolescent population. There is a rising incidence likely caused by an increased participation in youth sports [1–3]. The physis of the proximal tibia closes distally toward the tubercle apohysis. Which can lead to an avulsion injury during adolescence [4]. These fractures occur mostly during sports where there is forceful contraction of the quadriceps against resistance or with rapid knee flexion, for example sports with jumping and running motions [3,5]. The resulting fractures are most commonly classified by the Ogden classification. This classification expands upon the Watson-Jones system with modifiers ‘’A” and ‘’B” to indicate the displaced or comminuted fracture, respectively [6,7].

Even though these tibial tubercle fractures are rare, they can be accompanied with extensive soft tissue damage, periosteal stripping, vascular compromise, compartment syndrome and intra articular damage with possible growth plate damage [8–10]. The main age group associated with these kind of injuries are adolescents, who have still remaining bone growth. Therefore understanding its presentation and clinical outcome is essential. In current literature there are relatively few studies on these type of fractures and the subsequent treatment. However for the patient it is important that the clinician is able to inform about the proper treatment and possible outcomes. Therefore the aim of this study was to describe the patient characteristics, the treatment methods, clinical outcomes and complications of tibial tubercle fractures in children from the ages of 8–16 years.

### Patients and methods

A single center retrospective cohort study was conducted at a level 2 trauma center (Rijnstate hospital Arnhem), a teaching hospital in the Netherlands. This article was written according to the STROBE (Strengthening the Reporting of Observational Studies in Epidemiology) statement [11].

This study was approved by the Local Feasibility Committee of Rijnstate hospital Arnhem. All data were fully anonymized before we accessed them, and the ethics committee waived the requirement for informed consent. On March 4^th^ 2024, the data was accessed for research purposes. After data collection the data was anonymized.

### Patient selection

All the patients from this level 2 trauma center of 8–16 years of age with a tibial tubercle fracture, from January 2008 to October 2023 were considered for inclusion in the study. Patients with pre-existing bone diseases or neurological conditions, other bone fractures in the same extremity or a follow-up of less than 6 weeks were excluded from the analysis. Patient selection was performed searching the radiological report system using the following search strategy: ((“avulsion” OR “epifysiolysis” OR “fracture”) AND (“tuberosity”)) OR ((“avulsion” OR “epifysiolysis”) AND (“tibia”)). If the search term was found in the radiological report of performed medical imaging (X-ray, ultrasound, computed tomography (CT) scan or magnetic resonance imaging (MRI) scan), the imaging modality was viewed to see if a tibial tuberosity avulsion fracture was present.

### Patient and fracture characteristics

The patients’ medical records were screened to collect age, sex, body mass index (BMI), American Society of Anaesthesiologists (ASA) and relevant medical history consisting of comorbidities for example diabetes and endocrine disorders and mediation use like corticosteroids. Also the different kinds of diagnostic imaging were noted. The collected trauma and fracture variables included trauma mechanism, symptomatic side, open fracture and the classification using the Ogden classification. To assess the fracture classification, the radiographs from admission in the emergency room were used. The X-ray was used as primary basis for classification. If there was a CT or MRI available this was used as leading modality. To distinguish between subtypes of the Ogden classification, the following criteria were used. Type 1a is a simple epiphyseal separation with minimal displacement. Type 1b is a more severe epiphyseal separation with marked displacement. Type 2a is a Fracture through the metaphysis and physis, with no significant angulation or rotation. Type 2b is a more complex fracture through the metaphysis with some degree of angulation or displacement. Type 3a is a fracture that affects the growth plate but does not significantly disrupt the joint surface. Type 3b is a more complex fracture involving the growth plate and articular surface with severe displacement and joint instability. Two investigators (MM and DS) together with a radiologist (SJ) classified all fractures. Disagreement in classification was solved by re assessing the radiographs together. The opinion of the radiologist was decisive.

### Treatment and outcomes

The surgical variables collected included the interval between the trauma and surgery in days. The type of treatment was noted, surgery or cast. In case of surgery the different techniques of fixation were collected. In case of using cast as therapy, the duration of immobilisation was noted in weeks. The collected postoperative variables included the postoperative treatment protocol (weeks until mobilisation and weightbearing regime), the follow-up period of the patients, the return to sport ratio of the young patients and re-operation for removing the hardware. The return to sport outcome was defined by the use of the three-tier model, distinguishing between return to participation, return to sport and return to performance. This was recorded at the last follow-up appointment by the physician. All complications that occurred were recorded. The complications were defined as follows: wound-related complications included all wound healing disorders, superficial and deep wound infections. Implant-related complications that required revision surgery were categorized as inadequate fixation and/or implant breakage.

All variables were created before data extraction was performed. If variables were not noted in the electronic medical patient file they were considered as missing.

### Statistical analysis

IBM SPSS Statistics for Windows, Version 24.0, released 2016 (IBM Corp., Armonk,NY) was used for statistical analysis. Patient characteristics, medical history, fracture- and surgical data were presented using standard descriptive statistics expressed as numbers with percentages, means with standard deviation or as medians with interquartile range. Median and interquartile range was depicted in cases of non-normal distribution. To assess the distribution of continuous variables, the Kolmogorov-Smirnov test was performed, and histograms and boxplots were evaluated. To determine the interobserver reliability the intraclass correlation coefficient (ICC) was calculated using the single rating, absolute agreement, two-random effects model. The Fisher’s Exact Test was used to determine significant associations between outcomes in the surgical and nonsurgical group.

## Results

### Patient and injury characteristics

A total of forty-one TTFs in 40 patients, between the age of 8 and 16 years, treated from January 2008 to October 2023 were included. Patient and injury characteristics are presented in Table 1. Thirty-eight of these patients were male and 2 female. The mean age at injury was 14.3 years (range 8–16), the mean BMI 21.3 (range 16.6–32.6) and 95% (38/40) had a ASA I score. One of these patients had a comorbidity which might be associated with TTF, Osgood-Schlatter [12]. Another patient had Osteogenesis imperfecta which was not known at the time of injury. The other patients did not have any comorbidities which could be associated with known risk factors for TTFs. Of the 34 sports-related fractures, 25 (73.5%) occurred during soccer, representing 61% of the total cohort. Most fractures (n = 34; 85%) were diagnosed with an X-ray alone. There were no open fractures. The interobserver reliability was excellent, with a reliability coefficient of 0.93 and a 95% confidence interval ranging from 0.88 to 0.96.

**Table 1:** Patient and injury characteristics of all included patients with a tibial tubercle fracture.

Characteristics	All patients (n = 40)
Age at injury, years, mean (range)	14.3 (8 –16)
8–12 years	4 (10%)
12–16 years	36 (90%)
Sex	
Male	38 (95%)
Female	2 (5%)
BMI, mean (range)	21.3 (16.6–32.6)
<20	16 (40%)
20–25	13 (32.5%)
25–30	3 (7.5%)
>30	1 (2.5%)
Missing	8 (20%)
ASA score	
I	38 (95%)
II	2 (5%)
Comorbidities	
Osgood-Schlatter	1 (2.5%)
Osteogenesis imperfecta	1 (2.5%)
None	38 (95%)
Mechanism of injury	
Sport	34 (85%)
Soccer	25 (73.5%)
Traffic accident	2 (5%)
Fall injury	5 (12.5%)
Diagnostic technique	
X-ray	34 (85%)
X-ray and CT	5 (12.5%)
MRI	1 (2.5%)

#### Surgical treatment and outcomes.

Thirty-one patients, with thirty-two fractures receiving surgical treatment. The Ogden classification of these fractures was for 34.4% (11/32) IIa, 25% (8/32) IIb, 21.9% (7/32) IIIa, 12.5% (4/32) IIIb and for 6.3% (2/32) Ib. The duration of time between injury and surgery was for 59.4% (19/32) of the fractures within 24 hours (Table 2). Operative treatment of the fractures was achieved most commonly via open reduction and internal fixation with screw fixation, with the cannulated or cortical screws as most common. Also the use of cannulated or cortical screws combined with an anchor, a corkscrew and / or cerclage fixation was used in different patients. All the different surgical procedures are stated in Table 2. The mean duration of immobilisation after surgery was 39.3 days (range 14–42 days). Full weight bearing with a cast, splint or brace was applied after surgery for 71.9% (23/32 of the fractures. The other postoperative treatment regimens are stated in Table 2. The mean follow-up time for the surgically treated patients was 5.4 months (range 1.5–13 months). Four patients (each with one fracture) had a complication within 12 months after surgery.

**Table 2:** Surgical treatment of included tibial tubercle fractures.

Surgical treatment	Fractures (n = 32)
Ogden classification	
Ia	none
IIa	11 (34.4%)
IIIa	7 (21.9%)
Ib	2 (6.3%)
IIb	8 (25%)
IIIb	4 (12.5%)
Duration of time between injury and surgery	
<24 hours	19 (59.4%)
24–48 hours	4 (12.5%)
48–72 hours	1 (3.1%)
72–96 hours	3 (9.4%)
96–120 hours	2 (6.3%)
120–144 hours	2 (6.3%)
144–168 hours	1 (3.1%)
Surgical procedure	
Cannulated screws	11 (40.6%)
Singular cannulated screw	2 (6.3%)
Cannulated screws + anchors	1 (3.1%)
Cannulated screws + corkscrews	1 (3.1%)
Cannulated screws + anchors + cerclage	2 (6.2%)
Cortical screws	9 (28.1%)
Cortical screws + anchors	2 (6.3%)
Cortical screws + corkscrews	1 (3.1)
Cortical screws + anchors + cerclage	1 (3.1)
Spongiosa screws	1 (3.1%)
Singular spongiosa screw	1 (3.1%)
Postoperative weightbearing	
Full weight bearing with cast, splint or brace	23 (71.9%)
Non-weight bearing with cast, splint or brace	7 (21.9%)
50% of weight bearing with cast, splint or brace	2 (6.2%)
Complications during follow-up	
None	28 (87.5%)
Reoperation	3 (9.4%)
Readmission with no treatment	1 (3.1%)

The first was a refracture of the tibia tubercle 10 months after initial surgery, this happened spontaneously during a soccer game, there was no physical contact. The fracture was initially fixated with one cannulated screw. During the re operation it was fixated with four cortical screws and a drittelrohr plate. The second one, was a refracture of the tibia tubercle within 9 days after initial surgery, caused by a non-sport related new trauma. This fracture was initially fixated with one spongiosa screw, during the reoperation a cannulated screw and two cork screws were used. The third one had a spontaneously loosening of the singular cortical screw, 2.5 months after initial surgery. This screw was removed without new fixation, because of good bone consolidation. The fourth was a patient who was readmitted in the hospital three days after surgery, because of growing pain in the operated area and a temperature of 39.5 degrees Celsius. This patient was observed and recovered after two days without any antibiotic or surgical treatment. All these patients made a full recovery and returned to sport.

Twenty patients (64.5% (20/31)) had a reoperation for hardware removal. This was done after 12 months from initial surgery. At the end of the follow-up time twenty-nine patients (93.5% (29/31)) returned to sport. The follow-up time varied between patients.

### Nonsurgical treatment and outcomes

A total of nine patients (each with one fracture) were treated non-operative. The Ogden classification for these fractures was for 55.5% (5/9) Ia and for 44.4% (4/9) IIa. The duration of time between injury and diagnoses was for 66.7% (6/9) of the patients within 24 hours The other durations of time between injury and diagnoses are stated in Table 3. The non-operative treatment varied between non-weight bearing with splint, cast or immobilizer, 50% of weightbearing with splint, cast or immobilizer, or full weight bearing without splint, cast or immobilizer. All nonsurgical care regimens are stated in Table 3. The duration of immobilisation was six weeks for 77.8% (7/9) of the patients. The mean follow-up time of these patients was 3.6 months (range 0.5–12 months). There were no complications noted during the follow-up time. In total 77.8% (7/9) of the patients returned to sport at the end of the follow-up time.

**Table 3:** Nonsurgical treatment of included tibial tubercle fractures.

Nonsurgical treatment	Fractures (n = 9)
Ogden classification	
Ia	5 (55.5%)
IIa	4 (44.4%)
Duration of time between injury and diagnoses	
<24 hours	6 (66.7%)
48–72 hours	1 (11.1%)
144–168 hours	2 (22.2%)
Nonsurgical treatment	
Non-weight bearing	4 (44.4%)
50% weight bearing	4 (44.4%)
Full weight bearing without restrictions	1 (11.1%)
Duration of treatment	
6 weeks	7 (77.8%)
4 weeks	1 (11.1%)
Complications	none

## Discussion

Tibial tubercle fractures were often described as a rare injury in adolescents, with only 123 TTFs described prior to 1993 [9]. However recent studies have shown an increase of these injuries in children and adolescents for example Brey et al. [13] with almost 7 fractures per year between 2003 and 2010 and also Haber et al. [12] with 17 per year between 2011 and 2015. This increase may be caused by different factors like an increase in participation in high risk sports like soccer and basketball [8] and an increase in BMI with children and adolescents.

Although the number of children and adolescents with TTFs seems to be rising, there are relatively small case series in current literature. With two exceptions with Brey et al. with 48 patients (53 fractures) and Haber et al. with 228 (236 fractures) patients. This current study with 40 patients (41 fractures) can also be accounted as one of the relatively larger studies on this subject.

In this current study most of the TTFs occurred in adolescent males 95%, with a mean age of 14.3 years, this is comparable to current literature. Pretell-Mazzinin et al. [14] a relatively recent systematic review with studies dating back to 1970s, reported 97% of the fractures occurring in males with a mean age of 14.6 years. In other studies the percentage as Haber et al. and Brey et al. the percentages of male adolescents were 86% and 100% [12,13].

The lower incidence in adolescent females may be caused by the fact that females reach earlier skeletal maturity. With males having apophysis which close later in time and tending to have more quadriceps muscle mass. Which is perceived as a risk factor for this specific type of injury [15]. Another reason could be that in the past females were less represented in jumping and landing sports requiring high energy mechanism [16].

In this study Ogden type Ia and type IIa seem to be fractures which can be treated non operatively with a low complication rate, high return to sport ratio and good functional outcome. The total complication ratio was 9.8% (4/41) in the current study. All the complications occurred within patients with a higher Ogden classification which deemed surgical treatment. The percentage of refractures was 4.9% (2/41). This is comparable to the percentages in other studies with Haber et al. and Brey et al. both respectively 6%. The refractures in this current study occurred in patients who were fixated with a singular screw fixation. In hindsight there may be a suggestion that a singular fixation of the TTF may give a higher risk on a refracture. However the number of fractures included in this study are too small to determine a significant causal link. Other studies also described compartment syndrome as a complication, this did not occur in the current study population. Hardware removal was not included in the complication ratio in this study. However with 64.5% (20/31) of the patients undergoing a reoperation for removal this is relevant information for patient’s informed consent.

The surgical techniques that were used were mostly multiple cannulated or cortical screws. Also combinations were used with anchors, corkscrews and or cerclage. This is comparable to current literature. With Haber et al. [12] showing that the majority of the surgical procedures were performed with screws, but also in combination with plate and or tension band. Therefore these results suggest that there is no standardized best operative fixation method for TTF’s.

The return to sport ratio was in the surgical treated group 93.5% (29/31) and in the non-surgical treated group 77.8% (7/9). There was no significant difference between these two outcomes, p = 0.21. The return to sport ratio in the surgical treated group is comparable to current literature [12,13]. The return to sport ratio was determined at the end of the follow-up period, however this varied between all the patients. So there was no standardized cut off point.

The limitations of this study include that it is a retrospective single-centre study with no standardized treatment and follow-up protocol. Therefore this study may be prone to bias, a prospectively designed comparative study would be better suited. Also because the data was reviewed of one centre, it may be possible that patients with complications after surgery may have presented themselves in other hospitals. Another limitation is the possible bias caused by missing data. This was present in the variable BMI with 20% (8/40) of the data missing.

Another limitation was the use of only X-ray to determine the Ogden classification. This could lead to under classification of comminution which is better assessed on a CT scan. However the use of only X-ray with these type of fractures is regular practice in the Netherlands. Therefore there was no possibility to use a CT scan as leading imaging modality. Another limitation was the relatively short follow-up period. This was likely because most patients obtained a good radiograph and a full range of motion and returned to their activities after 3 months. Also the varying follow-up durations can be seen as a limitation. Specifically for the return to sport ratio. There was retrospectively no standardized cut-off point for assessing the return to sport for these patients. Therefore caution has to be advised with interpretation of these percentages and comparing it to current literature. Also the difference in follow-up duration between the surgical and non-surgical group can lead to confounding. Another limitation is the possible selection bias. By using a text-based search of the radiological reporting system there is a chance of missing patients whom radiology report did not match the specific terms used for extracting the data. However this bias was minimalized by using the surgical procedure coding. Despite several limitations we believe this study gives a good overview of patient characteristics and treatment options, surgical and non-surgical, for this relatively uncommon injury using a relatively large cohort. This can therefore be used by the clinician for making decisions together with the patient about the treatment of these kind of fractures.

### Conclusion

Tibial tubercle fractures in adolescents occur mostly in males during sports. The surgical treatment is not standardized and can be custom made per fracture. Patients receiving surgical or non-surgical treatment both have a good chance to return to sport after this specific injury.

## Supporting information

S1 File Supporting data in english. (XLSX)
