# Supplementary material for: Paediatric tibial tubercle fractures – A retrospective case series
Source: PLoS One. 2026 Aug 13;21(8):e0355958. doi: 10.1371/journal.pone.0355958 (PMC13472426; doi:10.1371/journal.pone.0355958)
Supplement: Supplementary file 1 [file pone.0355958.s002.pdf]

General Comments

This manuscript presents a retrospective case series of paediatric tibial tubercle fractures (TTFs) from a single level 2 trauma centre in the Netherlands, covering a 15-year period and including 40 patients (41 fractures). The topic is clinically relevant, the cohort is reasonably sized relative to the existing literature, and the authors are commended for providing a structured overview of patient characteristics, treatment approaches, and outcomes. The study addresses a genuine gap in the evidence base and has potential value for clinicians managing this uncommon injury.

However, several methodological, presentational, and ethical concerns must be addressed before the manuscript can be considered for publication. These are detailed below. Particular attention is drawn to issues surrounding fracture classification methodology, the handling of missing data, statistical analysis, the definition of key outcomes, and the adequacy of the ethics statement. Additionally, the manuscript requires a thorough revision for grammar and sentence structure throughout, as the current prose contains numerous errors that impair readability.

1. Grammar and Sentence Structure

The manuscript requires comprehensive revision for English language, grammar, and sentence construction. There are numerous errors throughout the text that impede clarity and, in some instances, create ambiguity about the intended meaning. While a full list of every error is beyond the scope of this review, the following examples are illustrative of the pervasive nature of the problem and are provided to guide revision:

- Background, lines 62–63: The sentence "The tibial tubercle fractures (TTFs) represent 3% of all proximal tibia fractures and less than 1% of all physeal fractures in the adolescent population, there is a rising incidence likely caused by an increased participation in youth sports". These should be separated into distinct sentences.
- Line 64: "a avulsion injury" should read "an avulsion injury."
- Line 64–65: "which during adolescence, a mechanically vulnerable period, can lead to a avulsion injury" is grammatically awkward and should be restructured for clarity.
- Line 75–76: "there are relative few studies" should read "there are relatively few studies."
- Line 173: "corckscrew" is a typographical error and should read "corkscrew."
- Line 258: "females reach earlies skeletal maturity" should read "females reach earlier skeletal maturity."
- Line 282: "this variated between all the patients" should read "this varied between patients."
- Lines 66–70: Several sentences in this paragraph are run-on constructions that would benefit from being broken up or restructured.

Overall the paper needs re-examining to check for grammar, spelling and sentence structure, as this would greatly benefit the readability of the work.

2. Fracture Classification Methodology

The authors describe their approach to Ogden classification as follows: two investigators (MM and DS) alongside a radiologist (SJ) independently classified all fractures, with disagreements resolved through discussion. While involving multiple classifiers is good practice, the description of this process is insufficiently detailed and methodologically incomplete in several respects.

First, no inter-rater reliability statistic is reported. For a study in which fracture classification is central to the reported outcomes, particularly the association between Ogden grade and treatment decision, it is essential to quantify the degree of agreement between classifiers prior to discussion-based resolution. A Cohen's kappa or similar statistic should be calculated and reported. Without this, readers cannot assess whether the classification was reliable or whether the final classifications are meaningful.

Second, and more fundamentally, the criteria used to assign fractures to specific Ogden subtypes are not described. The Ogden classification is a well-established system, but its application, particularly the distinction between subtypes (e.g., IIa versus IIb, or IIIa versus IIIb, where the "B" modifier denotes comminution), can be subjective, particularly on plain radiographs. The manuscript should specify which imaging modality was used as the primary basis for classification (the text states that 85% of fractures were diagnosed on X-ray alone, with CT used in a minority), and should describe the criteria applied when distinguishing between subtypes. Where CT was available, was this used preferentially for classification? If classification was performed on X-ray alone for the majority of cases, the potential for underclassification of comminution (which would be better appreciated on CT) should be acknowledged as a limitation.

Third, the resolution of disagreements "by discussion" is described without any further qualification. A more rigorous approach should either have been applied or should be described if it was applied. The authors should provide a more transparent account of this process.

3. Handling of Missing Data and the Assumption of Absence

In the Methods section, the authors state: *"If variables were not noted in the electronic medical patient file, we assumed that what was not recorded was absent for that patient."* This is a significant methodological assumption that is inadequately justified.

In retrospective studies drawing on clinical records, the absence of documentation does not reliably indicate the absence of a finding, symptom, or event. Clinical documentation is shaped by many factors unrelated to the actual clinical picture, including time pressure, documentation habits of individual clinicians, variation in institutional recording practices, and the perceived relevance of a given variable at the time of the clinical encounter. Assuming that an unrecorded variable was absent introduces a systematic bias that could affect multiple outcomes reported in this study, including complication rates, symptom documentation, and follow-up data.

This assumption is particularly consequential for the reported complication rate of 9.8%. If complications occurring outside the study centre (e.g., a patient presenting to a different hospital with a wound infection or vascular complication) were simply not recorded in the local electronic records, these would have been assumed absent under the authors' rule.

The authors acknowledge in the Discussion that patients with post-surgical complications may have presented at other institutions, but this acknowledgement does not adequately address the broader problem of the assumption as a methodological rule applied across all variables.

The authors should either provide a stronger methodological justification for this assumption, or, more appropriately, reclassify affected variables as missing and report the proportion of missing data per variable. Where missingness is substantial, sensitivity analyses or an explicit discussion of how missing data may have affected specific outcomes should be provided. At a minimum, the authors should present a table or supplementary material summarising the extent of missing data for each key variable, so that readers can assess the potential impact on the reported findings. The existing acknowledgement that BMI data were missing in 20% of patients is noted, but this is currently mentioned only implicitly in Table 1 and is not discussed in the limitations section.

4. Ambiguity in the Reporting of Soccer-Related Injuries

In the Results section (line 146–148), the authors state: *"Thirty-four fractures (82.9%) occurred during participating in sports, with 25 (73.5%) of them occurring during soccer."* The presentation of this statistic is ambiguous and risks misinterpretation.

The figure of 73.5% refers to the proportion of sports-related injuries attributable to soccer, that is, 25 out of 34 sporting injuries, and not to 73.5% of the total cohort of 41 fractures. As currently written, a reader may reasonably interpret this as meaning that 73.5% of all fractures in the cohort occurred during soccer, which would be an overstatement. In fact, 25 out of 41 fractures (approximately 61%) occurred during soccer when expressed as a proportion of the whole cohort.

The authors should revise this sentence to make the denominator explicit. A suggested revision would be: *"Of the 34 sports-related fractures, 25 (73.5%) occurred during soccer, representing 61% of the total cohort."* Similar care should be taken elsewhere in the manuscript where proportions are reported to ensure the denominator is always unambiguous.

5. Definition of Return to Sport

Return to sport is presented as a primary outcome of this study and is central to the conclusions drawn regarding the effectiveness of both surgical and non-surgical treatment. However, the manuscript does not provide a definition of what constitutes "return to sport," and this is a significant omission that substantially weakens the validity of this outcome.

Specifically, the following questions are left unanswered:

- Does return to sport mean return to any sporting activity, or return to the patient's pre-injury sport at a comparable level of intensity and participation? A patient who resumes light recreational activity may be classified as having returned to sport under a broad definition, while the same patient might not meet a stricter criterion requiring return to competitive participation at pre-injury level. These represent meaningfully different outcomes.

- At what time point is return to sport assessed? The manuscript states that return to sport was assessed "at the end of the follow-up period," but follow-up duration varied considerably between patients (range 1.5–13 months for surgical patients; 0.5–12 months for non-surgical patients). This means that the return-to-sport figure is not assessed at a standardised time point across the cohort. A patient with a 13-month follow-up has had far more opportunity to return to sport than one with a 1.5-month follow-up, yet both are included in the same percentage. This variability undermines any comparison of return-to-sport rates between groups or between this study and others in the literature.
- How was return to sport ascertained? Was it recorded at a clinical follow-up appointment, obtained by patient self-report, or derived retrospectively from clinical notes? The reliability of this ascertainment method should be described.

The authors should provide a clear, pre-specified definition of return to sport, ideally aligned with validated frameworks in the sports medicine literature (e.g., the three-tier model distinguishing return to participation, return to sport, and return to performance). If a standardised cut-off time point for assessment cannot be applied retrospectively, this must be clearly stated as a limitation, and the return-to-sport figures should be interpreted with explicit reference to the varying follow-up durations across the cohort.

6. Absence of Inferential Statistics

The manuscript relies exclusively on descriptive statistics. No inferential statistical comparisons are made between the surgical and non-surgical treatment groups, despite the fact that a comparison of outcomes between these groups, most notably return-to-sport rates (93.5% vs. 77.8%) and complication rates, is central to the conclusions of the paper.

The authors should either perform appropriate inferential statistical tests to compare key outcomes between groups, or provide an explicit and detailed justification for why such comparisons have not been made. Given the small sample size (32 fractures in the surgical group and 9 in the non-surgical group), standard parametric tests are likely inappropriate, but non-parametric alternatives or exact tests would be feasible. The authors should also consider whether the difference in follow-up duration between groups (mean 5.4 months surgical vs. 3.6 months non-surgical) constitutes a confounding factor that should be addressed.

If the authors' position is that the sample size is too small to support meaningful inferential comparisons, this must be stated explicitly in the Methods and Limitations sections, and the conclusions drawn from between-group comparisons must be appropriately tempered. The current conclusion that "patients receiving surgical or non-surgical treatment both have a good chance to return to sports after this specific injury" implies equivalence between groups that is not statistically supported by the data as presented.

7. Ethics Statement

The ethics statement as presented in both the submission form and within the manuscript body is insufficient for the purposes of publication in PLOS ONE and does not meet the standards expected for human participant research.

The current statement reads: *"In accordance with the legal department of the participating hospital and their local ethic commission, individual patient approval was not required."* While the waiver of individual patient consent is appropriate and acceptable for a retrospective anonymised study of this nature, the statement as written lacks the specific information required by PLOS ONE's ethics reporting guidelines. A declaration has been attached to the end of the manuscript, but this would be better placed within the methodologies. Reviewers and readers should not need to consult supplementary files to verify that a study met basic ethical requirements.

8. Limitations: Missing Data and Search Strategy

The limitations section of the Discussion addresses some weaknesses of the study, including its retrospective single-centre design and variable follow-up duration. However, two important limitations are either entirely absent or insufficiently discussed.

Missing variable data: As noted above in comment 3, BMI data were missing in 20% of patients. This is not acknowledged in the limitations section. More broadly, the implications of the assumption that unrecorded variables were absent, and the potential for this to have affected the reported complication rate, return-to-sport data, and other outcomes, should be discussed explicitly as a limitation.

Search strategy limitations: Patient identification was performed using a text-based search of the radiological reporting system. While the search terms used are described in the Methods, the authors do not acknowledge the inherent limitation of this approach: cases in which a tibial tubercle fracture was present but the radiological report did not use any of the specified search terms would have been missed entirely. This is a recognised limitation of text-based case identification in retrospective studies and could result in an underascertainment of cases, particularly for less severe fractures that may have been described using different terminology. The authors should acknowledge this as a potential source of selection bias and consider whether any supplementary search strategy (e.g., cross-referencing with surgical procedure coding or emergency department diagnostic codes) was used or could be described.
