# Supplementary material for: Paediatric tibial tubercle fractures – A retrospective case series
Source: PLoS One. 2026 Aug 13;21(8):e0355958. doi: 10.1371/journal.pone.0355958 (PMC13472426; doi:10.1371/journal.pone.0355958)
Supplement: Supplementary file 2 [file pone.0355958.s003.docx]

Manuscript number: PONE-D-25-56381

Article title: Paediatric tibial tubercle fractures – a retrospective case series

Dear Editor,

In response to your email we have made the requested changes in our manuscript and added the necessary files.

1. The Human participants Research Checklist is completed and added to our submission.
2. The participant consent was waived by the local ethics committee of Rijnstate Hospital. This is stated in the manuscript and their decision is added as file in to our submission.

Best regards,

Maarten Mosch

Orthopedic surgery Rijnstate hospital Arnhem
