# Supplementary material for: Paediatric tibial tubercle fractures – A retrospective case series
Source: PLoS One. 2026 Aug 13;21(8):e0355958. doi: 10.1371/journal.pone.0355958 (PMC13472426; doi:10.1371/journal.pone.0355958)
Supplement: Supplementary file 3 [file pone.0355958.s004.docx]

Manuscript number: PONE-D-25-56381

Article title: Paediatric tibial tubercle fractures – a retrospective case series

Dear Editor,

We would like to thank the reviewers for the comments on our paper. The answers to the reviewers’ comments are in red and can be found below after each separate remark. The manuscript has been revised according to the comments and we highlighted the changes in the revised manuscript. We hope you welcome our revision.

Best regards,

Maarten Mosch, on behalf of all co-authors

Orthopedic surgery Rijnstate hospital Arnhem

**Reviewer(s)' Comments to Author:**

1. **Grammar and Sentence Structure**

We agree with the reviewers and have checked for grammar, spelling and sentence structure throughout the manuscript.

2. **Fracture Classification Methodology**

First, no inter-rater reliability statistic is reported. For a study in which fracture classification is central to the reported outcomes, particularly the association between Ogden grade and treatment decision, it is essential to quantify the degree of agreement between classifiers prior to discussion-based resolution. A Cohen's kappa or similar statistic should be calculated and reported. Without this, readers cannot assess whether the classification was reliable or whether the final classifications are meaningful.

We agree with the reviewers and to assess the interobserver reliability we have used the interclass correlation coefficient:

*‘’Resulting in an excellent interobserver reliability of 0.93 with a 95% confidence interval ranging from 0.88 to 0.96’’*

Second, and more fundamentally, the criteria used to assign fractures to specific Ogden subtypes are not described. The Ogden classification is a well-established system, but its application, particularly the distinction between subtypes (e.g., IIa versus IIb, or IIIa versus IIIb, where the "B" modifier denotes comminution), can be subjective, particularly on plain radiographs. The manuscript should specify which imaging modality was used as the primary basis for classification (the text states that 85% of fractures were diagnosed on X-ray alone, with CT used in a minority), and should describe the criteria applied when distinguishing between subtypes. Where CT was available, was this used preferentially for classification? If classification was performed on X-ray alone for the majority of cases, the potential for underclassification of comminution (which would be better appreciated on CT) should be acknowledged as a limitation.

We agree with the reviewers and have elaborated about the distinction between subtypes and the used modalities in the method:

*The X-ray was used as primary basis for classification. If there was a CT or MRI available this was used as leading modality. To distinguish between subtypes of the Ogden classification, the following criteria were used. Type 1a is a simple epiphyseal separation with minimal displacement. Type 1b is a more severe epiphyseal separation with marked displacement. Type 2a is a Fracture through the metaphysis and physis, with no significant angulation or rotation. Type 2b is a more complex fracture through the metaphysis with some degree of angulation or displacement. Type 3a is a fracture that affects the growth plate but does not significantly disrupt the joint surface. Type 3b is a more complex fracture involving the growth plate and articular surface with severe displacement and joint instability. Two investigators (MM and DS) together with a radiologist (SJ) classified all fractures. Disagreement in classification was solved by re assessing the radiographs together.*

The use of only a x-ray is mentioned in the discussion as a limitation:

*‘’Another limitation was the use of only X-ray to determine the Ogden classification. This could lead to under classification of comminution which is better assessed on a CT scan. However the use of only X-ray with these type of fractures is regular practice in the Netherlands. Therefore there was no possibility to use a CT scan as leading modality’’*

Third, the resolution of disagreements "by discussion" is described without any further qualification. A more rigorous approach should either have been applied or should be described if it was applied. The authors should provide a more transparent account of this process.

This part is edited in the manuscript:

*‘’Disagreement in classification was solved by re assessing the radiographs together. The opinion of the radiologist was decisive.’’*

Thereby we want to mention that our interobserver reliability was excellent between the three observers.

3. **Handling of Missing Data and the Assumption of Absence**

In the Methods section, the authors state: "If variables were not noted in the electronic medical patient file, we assumed that what was not recorded was absent for that patient." This is a significant methodological assumption that is inadequately justified.

We agree with the reviewers and have altered the statement:

*‘’All variables were created before data extraction was performed. If variables were not noted in the electronic medical patient file they were considered as missing.’’*

The data missing only affected the variable BMI with 8 out of the 40 patients.

The other variables did not have any missing data.

This is as you say mentioned in our Table 1. We have now also assessed this in the discussion:

*‘’Another limitation is the possible bias caused by missing data. This was present in the variable BMI with 20% (8/40) of the data missing.’’*

4. Ambiguity in the Reporting of Soccer-Related Injuries

In the Results section (line 146–148), the authors state: "Thirty-four fractures (82.9%) occurred during participating in sports, with 25 (73.5%) of them occurring during soccer." The presentation of this statistic is ambiguous and risks misinterpretation.

We agree with the reviewers and have edited this into the manuscript:

*‘’Of the 34 sports-related fractures, 25 (73.5%) occurred during soccer, representing 61% of the total cohort.’’*

Also throughout the manuscript we have edited similar notations of percentages.

5. Definition of Return to Sport

The authors should provide a clear, pre-specified definition of return to sport, ideally aligned with validated frameworks in the sports medicine literature (e.g., the three-tier model distinguishing return to participation, return to sport, and return to performance). If a standardised cut-off time point for assessment cannot be applied retrospectively, this must be clearly stated as a limitation, and the return-to-sport figures should be interpreted with explicit reference to the varying follow-up durations across the cohort.

We agree with the reviewers and have edited the following definition and mentioned the not standardized cut off point:

*‘’The return to sport outcome was defined by the use of the three-tier model, distinguishing between return to participation, return to sport and return to performance. This was recorded at the last follow-up appointment by the physician.’’*

We agree with the reviewers that the variability in follow up interferes with the interpretation of the return to sport ratio. And also the comparison to current literature. We have edited this in the discussion:

*‘’Also the varying follow-up durations can be seen as a limitation. Specifically for the return to sport ratio. There was retrospectively no standardized cut-off point for assessing the return to sport for these patients. Therefore caution has to advised with interpretation of these percentages and comparing it to current literature*.’’

6. Absence of Inferential Statistics

The authors should also consider whether the difference in follow-up duration between groups (mean 5.4 months surgical vs. 3.6 months non-surgical) constitutes a confounding factor that should be addressed.

We agree with the reviewers that this factor could lead to confounding, which has now been described in the discussion:

*‘’Also the difference in follow-up duration between the surgical and non-surgical group can lead to confounding’’*

If the authors' position is that the sample size is too small to support meaningful inferential comparisons, this must be stated explicitly in the Methods and Limitations sections, and the conclusions drawn from between-group comparisons must be appropriately tempered. The current conclusion that "patients receiving surgical or non-surgical treatment both have a good chance to return to sports after this specific injury" implies equivalence between groups that is not statistically supported by the data as presented.

We agree with the authors and have used the Fishers’s exact test to analyze if there is a significant difference between return to sport ratio between the two groups:

It is not significant:

| **Chi-Square Tests** | | | | | |
| --- | --- | --- | --- | --- | --- |
|  | Value | df | Asymp. Sig. (2-sided) | Exact Sig. (2-sided) | Exact Sig. (1-sided) |
| Pearson Chi-Square | 1,928^a^ | 1 | ,165 |  |  |
| Continuity Correction^b^ | ,573 | 1 | ,449 |  |  |
| Likelihood Ratio | 1,640 | 1 | ,200 |  |  |
| Fisher's Exact Test |  |  |  | ,213 | ,213 |
| N of Valid Cases | 40 |  |  |  |  |
| a. 2 cells (50,0%) have expected count less than 5. The minimum expected count is ,90. | | | | | |
| b. Computed only for a 2x2 table | | | | | |

| **Risk Estimate** | | | |
| --- | --- | --- | --- |
|  | Value | 95% Confidence Interval | |
|  |  | Lower | Upper |
| Odds Ratio for Follow-up: return to sports? (no return to sport / yes return to sport) | 4,143 | ,494 | 34,746 |
| For cohort Type of treatment; 0. conservatief; 1. chirurgisch = conservatief | 2,571 | ,787 | 8,404 |
| For cohort Type of treatment; 0. conservatief; 1. chirurgisch = chirurgie | ,621 | ,230 | 1,676 |
| N of Valid Cases | 40 |  |  |

There was no significant difference between these two outcomes, p=0.21

7. Ethics Statement

The current statement reads: "In accordance with the legal department of the participating hospital and their local ethic commission, individual patient approval was not required." While the waiver of individual patient consent is appropriate and acceptable for a retrospective anonymised study of this nature, the statement as written lacks the specific information required by PLOS ONE's ethics reporting guidelines. A declaration has been attached to the end of the manuscript, but this would be better placed within the methodologies. Reviewers and readers should not need to consult supplementary files to verify that a study met basic ethical requirements.

We agree with the reviewers and have edited our ethics statement:

*‘’This study was approved by the Local Feasibility Committee of Rijnstate hospital Arnhem. All data were fully anonymized before we accessed them, and the ethics committee waived the requirement for informed consent.’’*

8. Limitations: Missing Data and Search Strategy

Missing variable data: As noted above in comment 3, BMI data were missing in 20% of patients. This is not acknowledged in the limitations section. More broadly, the implications of the assumption that unrecorded variables were absent, and the potential for this to have affected the reported complication rate, return-to-sport data, and other outcomes, should be discussed explicitly as a limitation.

As mentioned here above in point 3, the missing data of BMI is now mentioned as limitation in the discussion.

Search strategy limitations: Patient identification was performed using a text-based search of the radiological reporting system. While the search terms used are described in the Methods, the authors do not acknowledge the inherent limitation of this approach: cases in which a tibial tubercle fracture was present but the radiological report did not use any of the specified search terms would have been missed entirely. This is a recognised limitation of text-based case identification in retrospective studies and could result in an underascertainment of cases, particularly for less severe fractures that may have been described using different terminology. The authors should acknowledge this as a potential source of selection bias and consider whether any supplementary search strategy (e.g., cross-referencing with surgical procedure coding or emergency department diagnostic codes) was used or could be described

We agree with the reviewers and have edited the following:

*‘’Another limitation is the possible selection bias. By using a text-based search of the radiological reporting system there is a chance of missing patients whom radiology report did not match the specific terms used for extracting the data. However this bias was minimalized by using the surgical procedure coding’’*
