# Supplementary material for: Paediatric tibial tubercle fractures – A retrospective case series
Source: PLoS One. 2026 Aug 13;21(8):e0355958. doi: 10.1371/journal.pone.0355958 (PMC13472426; doi:10.1371/journal.pone.0355958)
Supplement: Peer review history

# Author comment: Author response to Decision Letter 0

**DOI:** 10.1371/journal.pone.0355958.r001

12 Nov 2025

Attachment Submitted filename: Response to editor.docx

# Peer review: Decision Letter 0

**DOI:** 10.1371/journal.pone.0355958.r002

**Authors:** Paolo Boffano

17 Mar 2026

Paediatric tibial tubercle fractures – a retrospective case series

PLOS One

Dear Dr. Mosch,

Thank you for submitting your manuscript to PLOS ONE. After careful consideration, we feel that it has merit but does not fully meet PLOS ONE’s publication criteria as it currently stands. Therefore, we invite you to submit a revised version of the manuscript that addresses the points raised during the review process.

==============================

**Please see reviewers' comments and suggestions.**

Please submit your revised manuscript by May 01 2026 11:59PM. If you will need more time than this to complete your revisions, please reply to this message or contact the journal office at plosone@plos.org. When you're ready to submit your revision, log on to https://www.editorialmanager.com/pone/ and select the 'Submissions Needing Revision' folder to locate your manuscript file.

- A letter that responds to each point raised by the academic editor and reviewer(s). You should upload this letter as a separate file labeled 'Response to Reviewers'.
- A marked-up copy of your manuscript that highlights changes made to the original version. You should upload this as a separate file labeled 'Revised Manuscript with Track Changes'.
- An unmarked version of your revised paper without tracked changes. You should upload this as a separate file labeled 'Manuscript'.

If you would like to make changes to your financial disclosure, please include your updated statement in your cover letter. Guidelines for resubmitting your figure files are available below the reviewer comments at the end of this letter.

If applicable, we recommend that you deposit your laboratory protocols in protocols.io to enhance the reproducibility of your results. Protocols.io assigns your protocol its own identifier (DOI) so that it can be cited independently in the future. For instructions see: https://journals.plos.org/plosone/s/submission-guidelines#loc-laboratory-protocols. Additionally, PLOS ONE offers an option for publishing peer-reviewed Lab Protocol articles, which describe protocols hosted on protocols.io. Read more information on sharing protocols at https://plos.org/protocols?utm_medium=editorial-email&utm_source=authorletters&utm_campaign=protocols.

We look forward to receiving your revised manuscript.

Kind regards,

Paolo Boffano

Academic Editor

PLOS One

Journal Requirements:

When submitting your revision, we need you to address these additional requirements.

1.Please ensure that your manuscript meets PLOS ONE's style requirements, including those for file naming. The PLOS ONE style templates can be found at

https://journals.plos.org/plosone/s/file?id=wjVg/PLOSOne_formatting_sample_main_body.pdf and

https://journals.plos.org/plosone/s/file?id=ba62/PLOSOne_formatting_sample_title_authors_affiliations.pdf

2. We note you have included a table to which you do not refer in the text of your manuscript. Please ensure that you refer to Table 3 in your text; if accepted, production will need this reference to link the reader to the Table.

3. Please include captions for your Supporting Information files at the end of your manuscript, and update any in-text citations to match accordingly. Please see our Supporting Information guidelines for more information: http://journals.plos.org/plosone/s/supporting-information.

4. If the reviewer comments include a recommendation to cite specific previously published works, please review and evaluate these publications to determine whether they are relevant and should be cited. There is no requirement to cite these works unless the editor has indicated otherwise.

Additional Editor Comments:

Please see reviewers' comments

[Note: HTML markup is below. Please do not edit.]

Reviewer's Responses to Questions

**Comments to the Author**

1. Is the manuscript technically sound, and do the data support the conclusions?

Reviewer #1: Partly

Reviewer #2: Partly

**********

2. Has the statistical analysis been performed appropriately and rigorously?

Reviewer #1: Yes

Reviewer #2: No

**********

3. Have the authors made all data underlying the findings in their manuscript fully available?

The PLOS Data policy

Reviewer #1: No

Reviewer #2: Yes

**********

4. Is the manuscript presented in an intelligible fashion and written in standard English?

Reviewer #1: Yes

Reviewer #2: No

**********

Reviewer #1: This manuscript presents a retrospective single-centre case series describing patient characteristics, treatment strategies, outcomes, and complications of paediatric tibial tubercle fractures. Given the rarity of this injury, the study addresses a clinically relevant topic and contributes additional observational data to the existing literature. The cohort size is reasonable for such an uncommon condition, and the manuscript is generally well structured and clearly written.

The study design is appropriate for the stated descriptive aim. Patient selection criteria are clearly defined, fracture classification was performed by multiple investigators, and outcomes are reported transparently using descriptive statistics. The authors appropriately acknowledge the limitations inherent to the retrospective design, including the lack of standardised treatment protocols and variable follow-up duration, and they avoid strong causal claims.

The statistical analysis is suitable for a descriptive case series, with appropriate use of summary statistics and assessment of data distribution. However, the absence of comparative or inferential analyses limits the strength of certain interpretative statements, particularly when discussing differences between surgical and non-surgical treatment outcomes. These observations should be interpreted cautiously and framed strictly as descriptive associations.

A relevant methodological concern is the assumption that unrecorded variables were considered absent, which may introduce information bias and should be discussed more explicitly as a limitation. In addition, while the Data Availability Statement indicates that data are included within the manuscript and supporting files, individual-level or de-identified raw data underlying the reported summary statistics are not fully accessible in a public repository. This does not fully comply with the PLOS Data Policy and should be clarified or addressed.

The manuscript is written in clear and intelligible scientific English. Minor grammatical issues and stylistic inconsistencies are present but do not impede understanding and can be addressed during revision.

Overall, this study provides a useful descriptive overview of paediatric tibial tubercle fractures and their management. With clarification regarding data availability and minor methodological and interpretative refinements, the manuscript would be suitable for publication.

Reviewer #2: Full review added as attachment.

Overall, paper has an interesting premise and the potential to provide useful clinical summary on a relatively obscure injury. However, significant revisions need to be made before publication.

**********

what does this mean?). If published, this will include your full peer review and any attached files.

If you choose “no”, your identity will remain anonymous but your review may still be made public.

**Do you want your identity to be public for this peer review?**  For information about this choice, including consent withdrawal, please see our Privacy Policy

Reviewer #1: No

Reviewer #2: No

**********

[NOTE: If reviewer comments were submitted as an attachment file, they will be attached to this email and accessible via the submission site. Please log into your account, locate the manuscript record, and check for the action link "View Attachments". If this link does not appear, there are no attachment files.]

To ensure your figures meet our technical requirements, please review our figure guidelines: https://journals.plos.org/plosone/s/figures

You may also use PLOS’s free figure tool, NAAS, to help you prepare publication quality figures: https://journals.plos.org/plosone/s/figures#loc-tools-for-figure-preparation.

NAAS will assess whether your figures meet our technical requirements by comparing each figure against our figure specifications.

Attachment Submitted filename: Paediatric tibial tubercle fractures - Review.pdf

# Author comment: Author response to Decision Letter 1

**DOI:** 10.1371/journal.pone.0355958.r003

9 May 2026

In the document response to reviewers we have responded to the comments of the reviewers.

Attachment Submitted filename: Response to reviewers_POS_MM2.docx

# Peer review: Decision Letter 1

**DOI:** 10.1371/journal.pone.0355958.r004

**Authors:** Paolo Boffano

29 Jul 2026

Paediatric tibial tubercle fractures – a retrospective case series

PONE-D-25-56381R1

Dear Dr. Mosch,

We’re pleased to inform you that your manuscript has been judged scientifically suitable for publication and will be formally accepted for publication once it meets all outstanding technical requirements.

Within one week, you’ll receive an e-mail detailing the required amendments. When these have been addressed, you’ll receive a formal acceptance letter and your manuscript will be scheduled for publication.

An invoice will be generated when your article is formally accepted. Please note, if your institution has a publishing partnership with PLOS and your article meets the relevant criteria, all or part of your publication costs will be covered. Please make sure your user information is up-to-date by logging into Editorial Manager at Editorial Manager® and clicking the ‘Update My Information' link at the top of the page. For questions related to billing, please contact billing support.

If your institution or institutions have a press office, please notify them about your upcoming paper to help maximize its impact. If they’ll be preparing press materials, please inform our press team as soon as possible -- no later than 48 hours after receiving the formal acceptance. Your manuscript will remain under strict press embargo until 2 pm Eastern Time on the date of publication. For more information, please contact onepress@plos.org.

Kind regards,

Paolo Boffano

Academic Editor

PLOS One

Additional Editor Comments (optional):

In agreement with the reviewer, I think that the Authors appropriately performed the suggested changes.

Reviewers' comments:

Reviewer's Responses to Questions

**Comments to the Author**

Reviewer #1: All comments have been addressed

**********

2. Is the manuscript technically sound, and do the data support the conclusions?

Reviewer #1: Yes

**********

3. Has the statistical analysis been performed appropriately and rigorously?

Reviewer #1: Yes

**********

4. Have the authors made all data underlying the findings in their manuscript fully available?

The PLOS Data policy

Reviewer #1: Yes

**********

5. Is the manuscript presented in an intelligible fashion and written in standard English?

Reviewer #1: Yes

**********

Reviewer #1: The authors have addressed the major methodological and reporting concerns raised in the previous review. The revised manuscript is substantially improved, particularly regarding fracture classification methodology, handling of missing data, definition of return-to-sport outcomes, statistical analysis, and clarification of study limitations.

The remaining issues are limited to minor language and stylistic inconsistencies that can be addressed during editorial processing. Overall, the manuscript is now suitable for publication.

**********

what does this mean?). If published, this will include your full peer review and any attached files.

If you choose “no”, your identity will remain anonymous but your review may still be made public.

**Do you want your identity to be public for this peer review?**  For information about this choice, including consent withdrawal, please see our Privacy Policy

Reviewer #1: No

**********

# Editor report: Acceptance letter

**DOI:** 10.1371/journal.pone.0355958.r005

**Authors:** Paolo Boffano

PONE-D-25-56381R1

PLOS One

Dear Dr. Mosch,

I'm pleased to inform you that your manuscript has been deemed suitable for publication in PLOS One. Congratulations! Your manuscript is now being handed over to our production team.

At this stage, our production department will prepare your paper for publication. This includes ensuring the following:

* All references, tables, and figures are properly cited

* All relevant supporting information is included in the manuscript submission,

* There are no issues that prevent the paper from being properly typeset

You will receive further instructions from the production team, including instructions on how to review your proof when it is ready. Please keep in mind that we are working through a large volume of accepted articles, so please give us a few days to review your paper and let you know the next and final steps.

Lastly, if your institution or institutions have a press office, please let them know about your upcoming paper now to help maximize its impact. If they'll be preparing press materials, please inform our press team within the next 48 hours. Your manuscript will remain under strict press embargo until 2 pm Eastern Time on the date of publication. For more information, please contact onepress@plos.org.

You will receive an invoice from PLOS for your publication fee after your manuscript has reached the completed accept phase. If you receive an email requesting payment before acceptance or for any other service, this may be a phishing scheme. Learn how to identify phishing emails and protect your accounts at https://explore.plos.org/phishing.

If we can help with anything else, please email us at customercare@plos.org.

Thank you for submitting your work to PLOS ONE and supporting open access.

Kind regards,

PLOS ONE Editorial Office Staff

on behalf of

Dr. Paolo Boffano

Academic Editor

PLOS One
